# Emergence of mosaic recombinant strains potentially associated with vaccine JXA1-R and predominant circulating strains of porcine reproductive and respiratory syndrome virus in different provinces of China

**DOI:** 10.1186/s12985-017-0735-3

**Published:** 2017-04-04

**Authors:** Huajian Zhao, Qinggong Han, Lei Zhang, Zhiyong Zhang, Yufeng Wu, Hong Shen, Ping Jiang

**Affiliations:** 1 Key Laboratory of Animal Diseases Diagnostic and Immunology, Ministry of Agriculture, College of Veterinary Medicine, Nanjing Agricultural University, Nanjing, 210095 China; 2 College of Life Sciences, Nanjing Agricultural University, Nanjing, 210095 China; 3 Bioinformatics Center, Nanjing Agricultural University, Nanjing, 210095 China; 4 College of Animal Science and Veterinary Medicine, Henan Institute of Science and Technology, Xinxiang, 453003 China

**Keywords:** PRRSV, Vaccine JXA1-R, Predominant circulating strains, Recombination

## Abstract

**Background:**

Porcine reproductive and respiratory syndrome virus (PRRSV) has caused several outbreaks in China since 2006. However, the genetic diversity of PRRSV in China has greatly increased by rapid evolution or recombination events. Modified live-attenuated vaccines are widely used to control this disease worldwide. Although the risk and inefficacy of the vaccine has been reported, the genetic diversity between epidemic field strains and vaccine strains in China has not been completely elucidated.

**Methods:**

A total of 293 clinical samples were collected from 72 pig farms in 16 provinces of China in 2015 for PRRSV detection. A total of 28 infected samples collected from 24 pig farms in nine provinces were further selected for immunohistochemical analysis and whole genome sequencing of PRRSV. Phylogenetic analysis and recombination screening were performed with the full genome sequences of the 28 strains and other 623 reference sequences of PRRSV.

**Results:**

Of 293 clinical samples, 117 (39.93%) were positive for PRRSV by RT-PCR. Phylogenetic results showed that the 28 strains were nested into sublineage 10.5 (classic highly pathogenic [HP]-PRRSV), sublineage 10.6 (HP-PRRSV-like strains and related recombinants), sublineage 10.7 (potential vaccine JXA1-R-like strains), and lineage 9 (NADC30-like strains and recombinants of NADC30-like strains), respectively, suggesting that multiple subgenotypes of PRRSV currently circulate in China. Recombination analyses showed that nine of 28 isolates and one isolate from other laboratory were potential complicated recombinants between the vaccine JXA1-R-like strains and predominant circulating strains.

**Conclusions:**

These results indicated an increase in recombination rates of PRRSV under current vaccination pressure and a more pressing situation for PRRSV eradication and control in China.

**Electronic supplementary material:**

The online version of this article (doi:10.1186/s12985-017-0735-3) contains supplementary material, which is available to authorized users.

## Background

Porcine reproductive and respiratory syndrome (PRRS) is an economically critical swine disease worldwide that causes severe reproductive failure in sows, poor viability of piglets, and respiratory disease with secondary infection in growing pigs. The agent of PRRS, PRRS virus (PRRSV), belongs to the *Arterivirus* genus and has a single-stranded positive-sense RNA genome of approximately 15 kb, which is organized into nine overlapping open reading frames (ORFs) [1–4]: ORF1a, ORF1b, ORF2a, ORF2b, and ORFs 3–7. ORF1a and ORF1b, which comprise 80% of the genome, encode the viral nonstructural proteins involved in genome transcription and replication [5], whereas ORF2a, ORF2b, and ORFs 3–7 encode the viral structural proteins GP2, E, GP3, GP4, GP5, M, and N, respectively [6–8]. The two genotypes of PRRSV, the European type (Type 1) and North American type (Type 2), share only about 60% nucleotide similarities at the genomic level and are represented by Lelystad virus and VR-2332, respectively [9–11].

The North American type PRRSV has been a major viral genotype in China [12–14], but was not a major concern until 2006, when highly pathogenic PRRSV (HP-PRRSV), characterized by a 30-aa deletion in Nsp2, emerged [15]. Having evolved from a less-pathogenic variant in China, HP-PRRSV has widely spread and continues to cause high mortality and huge economic losses to the swine industry in China [15, 16]. In a second major outbreak in China and other Southeast Asian countries in 2009 and 2010, the HP-PRRSV strain was later shown to be a recombinant between two Chinese PRRSV strains [17, 18]. NADC30 was isolated in the US in 2008 and in China in 2013 [19]. Associated with the importation of NADC30-like strains or recombination events between NADC30-like and domestic strains in China, a third outbreak occurred from 2013 to 2015 [19–21].

Imported and domestic modified live-attenuated vaccines (MLVs) have been widely used to safeguard against PRRSVs since the outbreaks in China. However, evidence of the risk and inefficacy of MLVs to prevent the spread of the latest PRRSV strain has begun to accumulate [21–24]. In order to investigate the epidemiological and evolutionary characteristics of PRRSV in 2015, a total of 293 clinical samples were collected from pigs in 16 provinces in China and the full-length sequence of 28 PRRSV isolates were sequenced and analyzed.

## Methods

### Sample collection

A total of 293 clinical lung samples were collected from pigs from different swine herds that experienced high fever and reproductive and respiratory syndrome in 16 provinces of China in 2015. A portion of each sample was homogenized for RNA extraction and stored at −70 °C. Meanwhile, the clinical symptoms, morbidity, mortality, and changes in autopsy tissues of pigs were examined.

### RNA extraction and reverse transcriptase polymerase chain reaction (RT-PCR)

Total RNA was extracted from tissue homogenates using TRIzol reagent (Life Technologies, New York, NY, USA). cDNA was constructed from 7 μL of RNA using M-MLV reverse transcriptase (Promega, Madison, WI, USA). Two microliters of cDNA was used as a template for subsequent PCR analysis. Specific PRRSV nucleotide sequences of 143 bp were amplified with the primers 5′-AGC TGT GCC ARA TGY TGG-3′ and 5′-GGT RAA GTG ATG YCT GAC-3′ targeting ORF7. The cycling conditions were 95 °C for 1 min, followed by 35 cycles at 95 °C for 30 s, 56 °C for 30 s, 72 °C for 30 s, and a final extension at 72 °C for 2 min.

### Immunohistochemical (IHC) analysis

The presence of PRRSV antigens in lung tissues was examined by IHC analysis. Briefly, the lung tissues were fixed in 10% neutral buffered formalin for 48 h, sectioned, and stained with monoclonal antibodies specific for the N protein of PRRSV for detection of antigen-positive cells, as previously described [25].

### Full-length sequencing of PRRSV

The 28 PRRSV infected samples (Table 1) were chosen for further whole genome sequencing. Viral RNA purification from lung tissues and cDNA synthesis were performed as described previously [26], and PCR amplification of 14 fragments covering the PRRSV genome was performed with primers described in a previous report with minor modifications [27] (Additional file 1: Table S1). The quality of the PCR products was confirmed by electrophoresis on a 1% agarose gel and then purified using the PureLink Quick Gel Extraction Kit (Life Technologies). Then, the PCR products were quantified, cloned into the pMD18-T vector (Takara Bio, Inc., Shiga, Japan), and sequenced with an automated genome sequencer (Genetic Analyzer 3730XL; Applied Biosystems, Foster City, CA, USA).

**Table 1:** Information of the 28 PRRSV isolates in China

No.	Isolate	Province	Accession no.	Clinical background		Herd size(sows)	Vaccination history of MLV
				Morbidity (%)	Mortality (%)		
1	15GD1	Guangdong	KX815407	40.2	21.4	700	No
2	15GD2	Guangdong	KX815408	63.6	62.9	3000	JXA1-R,TJM-F92
3	15GD3	Guangdong	KX815409				
4	15GD4	Guangdong	KX815410	41.6	7.2	2400	No
5	15HEB1	HeBei	KX815411	80.8	86.5	1400	No
6	15HEB3	HeBei	KX815412	62.7	53.4	450	JXA1-R
7	15HEN1	Henan	KX815413	71.7	72.5	1000	No
8	15HEN3	Henan	KX815414	29.6	3.4	1300	No
9	15HEN4	Henan	KX815415	33.2	7.6	420	No
10	15HUN1	Hunan	KX815416	65.4	54.1	3400	No
11	15HUN2	Hunan	KX815417				
12	15HUN3	Hunan	KX815418	23	61.4	200	R98
13	15JX1	Jiangxi	KX815419	80	48.8	700	No
14	15JX2	Jiangxi	KX815420	67.4	26.6	390	No
15	15JX3	Jiangxi	KX815421	2.9	2.2	400	No
16	15JX4	Jiangxi	KX815422	12.3	54.9	1500	RespPRRS
17	15LN1	Liaoning	KX815423	51.1	23.8	320	No
18	15LN2	Liaoning	KX815424	35.4	2.3	250	No
19	15LN3	Liaoning	KX815425	94.7	87.1	600	No
20	15SC1	Sichuan	KX815426	12.9	56.2	500	JXA1-R
21	15SC2	Sichuan	KX815427	33.8	4.8	800	No
22	15SC3	Sichuan	KX815428	75	90.8	1500	No
23	15SN1	Shaanxi	KX815429	50.6	31.6	350	TJM-F92
24	15SN2	Shaanxi	KX815430				
25	15SN3	Shaanxi	KX815431				
26	15ZJ1	Zhejiang	KX815432	28.4	16	660	CH-1R
27	15ZJ2	Zhejiang	KX815433	65	35.9	1400	JXA1-R
28	15ZJ3	Zhejiang	KX815434	57.6	32.3	570	No

### Phylogenetic analysis

A total of 651 genomic sequences, which included 28 sequences from the present study and all available sequences of type 2 PRRSVs (*n* = 623) retrieved from the GenBank database in March 2017 (https://www.ncbi.nlm.nih.gov/genbank/) were aligned using the MUSCLE multiple alignment program (v3.8.31) [28] under default settings with minor manual adjustments afterward. Phylogenetic trees were constructed using the neighbor-joining algorithm with the Kimura 2-parameter substitution model and bootstrap tests of 1000 replicates using the MEGA6 sequence alignment tool [29]. Interactive tree of life (http://itol.embl.de) [30] was employed to display and annotate the phylogenetic trees. The genetic distances of intra- and inter- clusters were computed using MEGA 6 with Kimura 2-parameter model and 1000 bootstrap replicates. Sub-data sets with a diversity level of >10% were further divided into smaller monophyletic clusters and sublineages within lineages were identified with a cut off of 1.4%.

### Recombination analyses

To accurately identify potential recombinants, sequences of the 28 strains identified in this study were combined with up to 623 isolate sequences for recombination screening. Recombination events were only considered significant when supported by at least three of the following five methods: RDP [31], GENECONV [32], MAXCHI [33], CHIMAERA [34], and 3SEQ [35] implemented in RDP v4.16 [36]. In order to check the recombination signals and estimate the breakpoint locations, sequences of potential recombinants isolated in this study or similar sequences of the parental lineage and those retrieved from the GenBank database were chosen for similarity plot analysis implemented in SIMPLOT v3.5.1 [37], with a window of 500 bp and step size of 10 bp. Furthermore, recombination events were confirmed with a neighbor-joining phylogenetic tree.

Only the strains that fit all of the following three criteria, as modified from a previous report [23], were recognized as JXA1-R (JXA1 P80)-like strains: (1) nested in the phylogenetic tree within the same cluster as the vaccine strain JXA1-R, (2) sharing the highest sequence identity with the JXA1-R strain (>99%), and (3) presenting amino acids (aa) unique to JXA1-R strain derivatives.

## Results

### Detection of PRRSV in the clinical samples

**Fig. 1:**
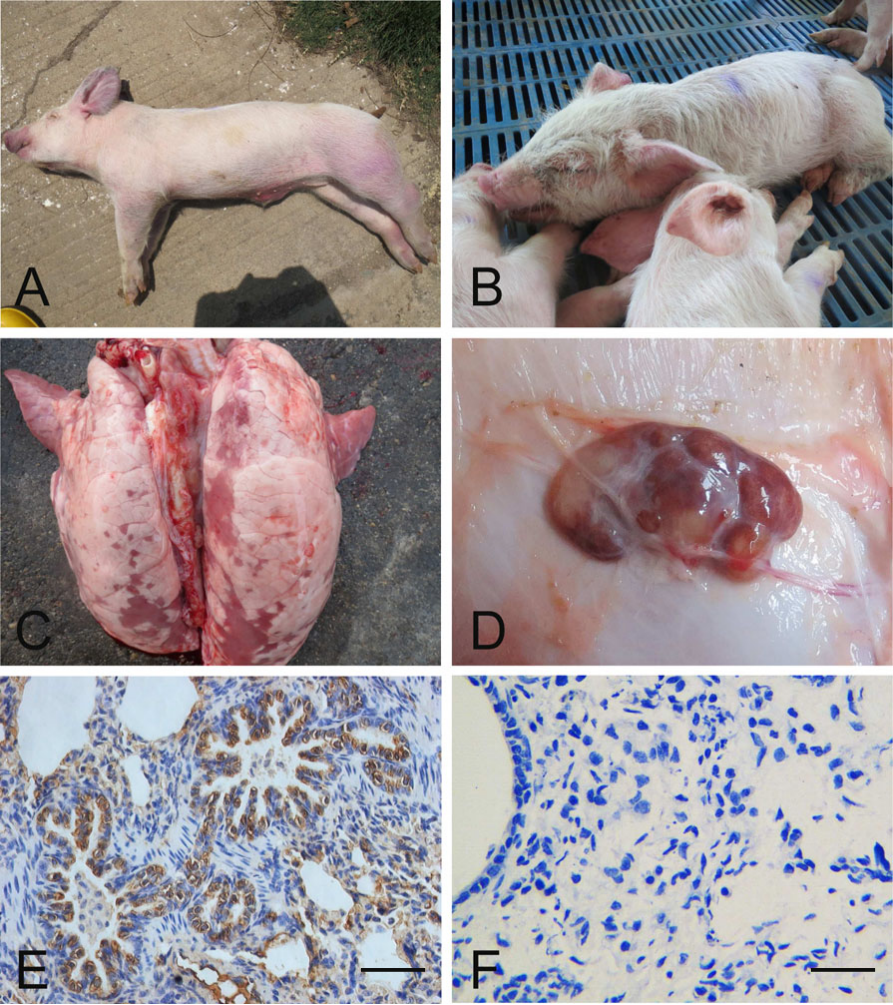
Clinical symptoms, gross lesions, and PRRSV detection by immunohistochemical analysis. The representative sick pigs exhibited *blue ears*, *blue noses* (**a**), and conjunctivitis (**b**); hyperplasia and edema in the lungs (**c**), and hemorrhagic spots in the lymph nodes (**d**). There were obvious PRRSV antigens in the lung tissue of diseased piglets (**e**) as compared to healthy piglets (**f**). Bar = 50 μm

A total of 293 clinical samples were collected from different pig farms in 16 provinces in China in 2015. Of these samples, 117 (39.93%) were positive for PRRSV detection by RT-PCR, which was similar to that report in 2010 (45.2%) [17], suggesting that PRRSV was still prevalent in China in 2015. Representative sick pigs exhibited high and continuous fever, labored breathing, blue ears, blue nose, erythematous blanching rashes, pimples in the ears or on the back, and loss of appetite (Fig. 1a, b). Pathological examination results showed hyperplasia, edema, and hemorrhage in the lungs (Fig. 1c), and severe hemorrhagic spots in the lymph nodes (Fig. 1d). IHC results indicated obvious PRRSV antigen in the lungs of diseased piglets (Fig. 1e), as compared with healthy piglets (Fig. 1f).

The full length sequences of 28 PRRSV strains from 24 pig farms in nine provinces were amplified and sequenced for further analysis. Strain names, areas, accession numbers, and clinical backgrounds are summarized in Table 1.

### Phylogenetic analysis

**Fig. 2:**
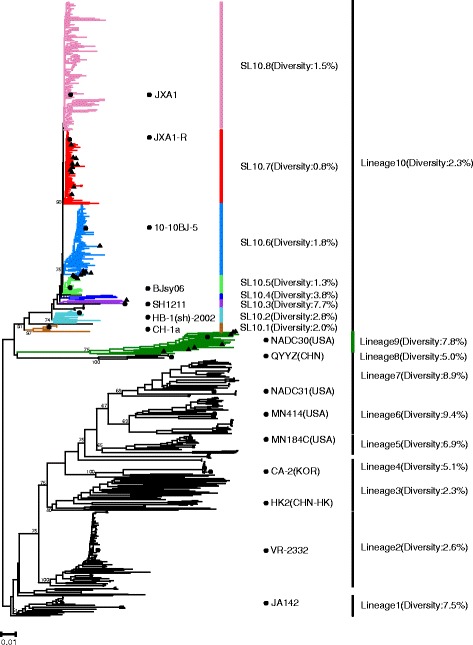
Phylogenetic tree of 28 strains isolated in this study and other 623 PRRSV strains. Lineage 9 is highlighted in *green*. Sublineages 10.1–10.8 are highlighted in *sandy brown*, *light blue*, *purple*, *blue*, *light green*, *deep sky blue*, *red*, and *pink*, respectively. The representative strains in lineages or sublineages are labeled with *black circles* (●) and the 28 strains isolated in this study are labeled with *black triangles* (▲). In sublineage 10.7, 10 isolates (15ZJ2, 15ZJ3, 15HUN3, 15LN2, 15JX2, 15JX3, 15JX4, 15HEN3, 15SC1, and 15SC2) from the present study were identified as potential vaccine JXA1-R-like strains with a 30-aa deletion in Nsp2

As shown in Fig. 2, the phylogenetic tree showed that the 28 isolates and another 623 isolates of PRRSV were clustered into 10 lineages, which generally have intralineage diversities of less than 10% (Table 2). The pairwise homology of the 28 isolates ranged from 76.3 to 99.9%, and the 28 isolates were divided into two lineages: 9 and 10 (Additional file 2: Table S2; Fig. 2). Meanwhile, the vaccine strain (ATP) and its parental strain (JA142) belonged to lineage 1. MLV RespPRRS/Repro and its parental, the prototype north American strains VR-2332, belonged to lineage 2. Some local endemic strains in Hong Kong and Taiwan, like isolates HK2, were clustered into lineage 3, and isolate CA-2, a virulent Korean strain, was clustered into lineage 4. MN184 variants, virulent MN414, and NADC31 were nested within lineage 5, 6, and 7, respectively. QYYZ-like strains and it recombinants isolated from southern China were clustered into lineage 8.

**Table 2:** Pairwise interlineage genetic distance comparisons for complete genome sequences of type 2 PRRSVs

	% Difference for lineage									
Lineage	1	2	3	4	5	6	7	8	9	10
1										
2	10.8									
3	17.4	14.9								
4	18.3	15.4	19.7							
5	17.7	16.0	19.5	16.8						
6	19.1	17.9	20.9	20.6	15.2					
7	19.9	18.5	21.3	18.9	12.9	13.4				
8	15.8	14.1	18.7	20.8	20.5	21.9	21.9			
9	18.0	16.9	20.8	20.5	16.2	13.2	16.6	21.1		
10	10.6	11.8	18.3	18.8	18.7	20.1	20.3	13.9	18.5	

Seven strains (15LN1, 15ZJ1, 15HEN4, 15JX1, 15HEN1, 15SC3, and 15LN3) in this study were nested in lineage 9 together with NADC30-like strains and its recombinants. A total of 350 isolates mainly from China were clustered into lineage 10, which was divided into eight sublineages (10.1–10.8).

CH-1a (isolated in 1996) was located in sublineage 10.1 and HB-1(sh)/2002 (isolated in 2002) was nested in sublineage 10.2. 15HEB1 isolated in this study and recombinant SH1211 were nested within sublineage 10.3. The early HP-PRRSV isolates [15, 26] were clustered in sublineage 10.5 and 10.8. Four strains (15SN1, 15SN2, 15SN3, and 15GD4) isolated in this study and BJsy06 (isolated in 2006) were located in sublineage 10.5. HP-PRRSV JXA1, isolated in the first outbreak in 2006 [15], was clustered in subgroup 10.8. The isolates identified in this study (15GD2, 15GD3, 15HUN1, and 15HUN2) were potential recombinants associated with 2009–2010 HP-PRRSV-like strains and were located in sublineage 10.6. Sublineage 10.7 consisted of JXA1 derivatives by serial passaging in MARC-145 cells [38, 39] and potential vaccine JXA1-R (JXA1 P80)-like strains. In sublineage 10.7, 10 isolates (15ZJ2, 15ZJ3, 15HUN3, 15LN2, 15JX2, 15JX3, 15JX4, 15HEN3, 15SC1, and 15SC2) from the present study were identified as potential vaccine JXA1-R-like strains with a 30-aa deletion in Nsp2.

These results indicated that multiple subgenotypes of PRRSV co-existed in China in 2015, which included classic HP-PRRSV (sublineages 10.5 and 10.8), 2009–2010 HP-PRRSV-like strains and its recombinants (sublineage 10.6), potential vaccine JXA1-R-like strains (sublineage 10.7), and NADC30-like strains and its recombinants strains (lineage 9).

### Distribution of JXA1-R vaccine-like strains in China

**Fig. 3:**
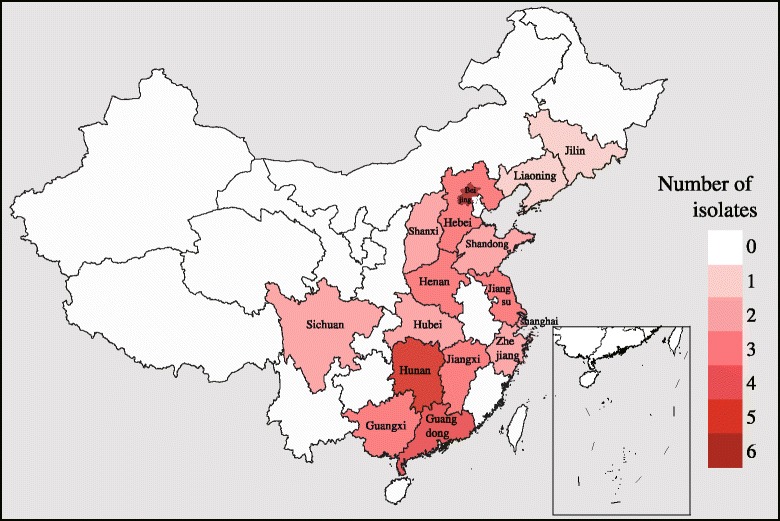
Distribution of 44 potential JXA1-R-like strains in China. Distribution if presented as the number of isolates per province or autonomous city (region), from most cases (6, *dark red*) to no case (0, *white*)

In sublineage 10.7 with vaccine strain JXA1-R as shown in Fig. 2, there were 44 JXA1-R-like strains, which included the 10 potential JXA1-R-like strains in this study and seven reported strains in China (NT1, NT2, NT3, 11NZ-GD, 11XX-GD, 11SH1-GD, and 11SH-GD) [23, 27]. These 27 Chinese strains were deposited in the GenBank database because they shared the highest nucleotide identities with JXA1-R strains (>99%) (Additional file 3: Table S3) and contained 6–11 amino acids unique to JXA1-R (Additional file 4: Figure S1). The geographical distribution results revealed that those potential JXA1-R-like strains were detected in all 16 provinces or autonomous cities (regions) with a wide geographical range in China (Fig. 3).

### Recombination analyses

**Fig. 4:**
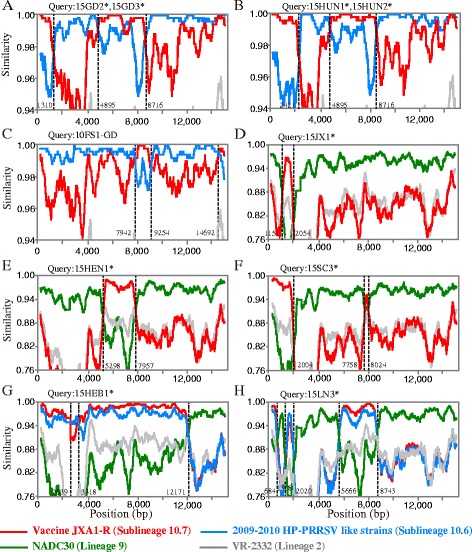
SimPlots for all putative recombinants analyzed in this study. Comparisons of genetic similarity between recombinant and parental strains were made using SimPlot. The results are shown for strains 15GD2*, 15GD3* (**a**), 15HUN1*, 15HUN2* (**b**), 10FS1-GD (**c**), 15JX1* (**d**), 15HEN1* (**e**), 15SC3* (**f**), 15HEB1* (**g**), and 15LN3* (**h**). Strains isolated in this study are marked with asterisks (*). The *vertical axis* represents the nucleotide sequence similarity between the putative recombinant strains, vaccine JXA1-R (sublineage 10.7, *red*), 2009-2010 HP-PRRSV-like strains (sublineage 10.6, *deep sky blue*), strain NADC30 (lineage 9, *green*), and strain VR-2332 (lineage 2, *grey*). The specific parental 2009-2010 HP-PRRSV strains of the recombinant strains are shown in Table 3. The *horizontal axis* shows the relative nucleotide position in reference to the full-length genome of VR-2332. Recombination breakpoints are shown as *black dotted lines* with the location indicated at the bottom

**Fig. 5:**
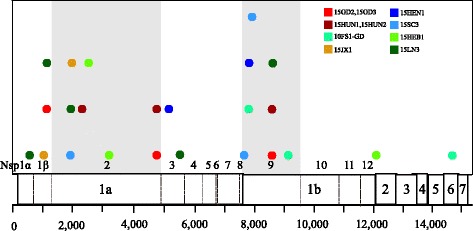
Recombination breakpoints identified across the full-length genome. The putative recombinants are listed in the key, with the colored lines matching each corresponding breakpoint. Most breakpoints were localized in Nsp2 and Nsp9, and the backgrounds of the two regions are highlighted in *grey*

All recombination events in RDP were detected and confirmed by SIMPLOT and phylogenetic trees (Additional file 5: Figure S2). As shown in Fig. 4, nine of 28 isolates and one isolates from another laboratory were the result of recombination events between the JXA1-R and predominant circulating strains. Three kinds of recombination events were involved: inner-lineage, inter-lineage, and both inner-lineage and inter-lineage (Table 3). First, 15GD2, 15GD3, 15HUN1, 15HUN2, and 10FS1-GD were the products of inner-lineage recombination events between 2009 and 2010 HP-PRRSV-like strains (sublineage 10.6) and JXA1-R vaccine strains (sublineage 10.7). The genomes of 15GD2 and 15GD3 shared the same recombination pattern, as did the genomes of 15HUN1 and 15HUN2. Second, 15JX1, 15HEN1, and 15SC3 were inter-lineage recombination events between strains NADC30 (lineage 9) and JXA1-R. Third, 15HEB1, and 15LN3 were mosaic recombination events between strains NADC30, 2009–2010 HP-PRRSV, and JXA1-R. These results indicate that the recombination events in China were relatively complicated. Among them, Nsp2 or Nsp9 exhibited the complicated recombination events, involving eight breakpoints from six recombination events (Fig. 5).

**Table 3:** PRRSV recombination events documented in this study

Recombination set	Year	Location	Parental strains	Breakpoint position (nt)
**Set1 (** **innerlineages** **)**				
**(** **SL10.7** **±** **10.6** **)**				
15GD2*,15GD3*	2015	Guangdong	15GD1 + JXA1-R	1310, 4895, 8716
15HUN1*,15HUN2*	2015	Hunan	15GD1 + JXA1-R	2421, 4895, 8716
10FS1-GD	2010	Guangdong	10HD-GD + JXA1-R	7942, 9254, 14692
**Set2 (** **interlineages** **)**				
**(** **L9** **±** **SL10.7** **)**				
15JX1*	2015	Jiangxi	NADC30 + JXA1-R	1151, 2054
15HEN1*	2015	Henan	NADC30 + JXA1-R	5298, 7957
15SC3*	2015	Sichuan	NADC30 + JXA1-R	2004, 7758, 8024
**Set3 (** **inner & interlineages** **)**				
**(** **L9** **±** **SL10.7** **±** **10.6** **)**				
15HEB1*	2015	Hebei	NADC30 + JXA1-R + 10-10JX	2639, 3318, 12171
15LN3*	2015	Liaoning	NADC30 + JXA1-R + 10-10JX	684, 1318, 2020, 5666, 8743

*Strains isolated in this study; Breakpoint position in reference to VR-2332 (accession number U87392)

## Discussion

PRRSV is among the most economically important viruses in the swine industry worldwide [40, 41]. In 2006, a HP-PRRSV with a unique discontinuous 30-aa deletion in Nsp2 emerged and caused the most severe viral diseases in swine in China to date [15]. The genetic diversity of this HP-PRRSV has greatly increased by rapid evolution and recombination events [14, 17–19]. The current commercial PRRSV vaccines cannot provide complete protection against this disease [21]. In this study, 117 (39.93%) of 293 clinical samples were positive for PRRSV by RT-PCR, similar to that reported in 2010 (45.2%) [17]. Full-length sequences of 28 PRRSV isolates showed that they belonged to two lineages together with HP-PRRSV isolated in China. Among them, 10 JXA1-R-like isolates and 44 other Chinese strains were possibly derived from JXA1-R strains by recombination events of different strains. These findings indicated that the mosaic recombinant strains associated with the vaccine strains JXA1-R and HP-PRRSV were the predominant circulating strains of PRRSV in China.

The virulence reversion of the vaccine strain JXA1-R has been described [23, 27], but no recombination of this strain has yet been reported. Recombination analyses showed that 10 strains, which included nine strains from the present study and one isolate retrieved from the GenBank database, evolved through complicated recombination events between vaccine JXA1-R-like strains and predominant circulating strains in China. Phylogenetic analysis showed that the nine strains isolated in this study were positioned within major clusters with recombinants from other laboratories (Fig. 2). Furthermore, all breakpoints of the recombinants isolated in this study were not adjacent to the corresponding primer binding sites, suggesting that all of the recombinant strains isolated in this study are circulating recombinant strains rather than laboratory artifacts.

Most of the breakpoints of recombination events in this study were located in Nsp2 or Nsp9. Similarly, Liu et al. [11] conducted in vivo experiments to identify recombination events with Nsp2, ORF3, and ORF5, which demonstrated that Nsp2 was the most complicated region for recombination. Furthermore, all breakpoints of the recombinant strain Em2007 (lineage 10) were located in Nsp2 or Nsp9 [22], as were two of the three breakpoints of 2009–2010 HP-PRRSV [18]. Thus, it is possible that Nsp2 or Nsp9 is the hot region for breakpoint of recombination events.

Geographically, previous vaccine-like strains or recombinants related with vaccine in China were located in limited ranges and represented by single sequences. However, the results of the present study revealed that JXA1-R-like strains were detected in all 16 provinces or autonomous cities (regions) in China, denoting a wide geographical range. In 2010, only two JXA1-R-like strains (GX1002 or GX1003) were examined. Since then, the emergence of JXA1-R-like strains has greatly increased, as shown by the data presented in Table 4. These findings imply that HP-PRRSV vaccine-like strains were the predominant strains circulating in the field followed by the PRRSV vaccination.

**Table 4:** Time of emergence of vaccine JXA1-R-like strains in China

Isolation year	No. Isolation	Isolate	Reference
2010	2	GX1002, GX1003	[45], GenBank
2011	6	GX1001, NVDC-HeB1-2011, 11NZ-GD, 11SH1-GD, 11SH-GD, 11XX-GD	[27, 46], GenBank
2012	10	NT1, NT2, NT3, JL-04/12, NVDC-BJ3-2012, NVDC-BJ4-2012, NVDC-BJ5-2012, NVDC-BJ6-2012, NVDC-BJ9-2012, NVDC-SD2-2012	[23, 47], GenBank
2013	7	NVDC-SDXX-2013, NVDC-HBCZ-2013, NVDC-SXJC-2013, HNyc13, HEB-2013, HEB 20130008-14, NVDC-BJPG-2013	GenBank
2014	8	HNxa14, HUN-2014, HB2014001, HENZK-1, NVDC-shh01-2014, NVDC-SHH02-2014, NVDC-HuNCS-2014, NVDC-13SXJC-2014	GenBank
2015	11	HENPDS-2, 15ZJ2, 15ZJ3, 15HUN3, 15LN2, 15JX2, 15JX3, 15JX4, 15HEN3, 15SC1, 15SC2	GenBank, this study

Recombination events between field PRRSV strains could result in greater PRRSV pathogenicity, such as strain JL580 from the recombination between NADC30 and HP-PRRSV 09HEN1, which has been confirmed as highly pathogenic [19]. In this study, the PRRSV-positive piglets exhibited severe clinical symptoms and gross lesions typical of HP-PRRS. Morbidity (63.6%–94.7%) and mortality (48.8%–90.8%) rates were relatively high among piglets infected the recombinant strains (Table 1, Additional file 6: Figure S3), suggesting that the recombinant strains associated with JXA1-R might be highly pathogenic. Of course, the pathogenicity of these recombinant JXA1-R-like strains should be examined in future studies.

In order to control this disease, at least seven MLV PRRSV vaccine strains (JXA1-R, HuN4-F112, TJM-F92, GDr180, RespPRRS/Repro, CH-1R, and R98) have been widely used in China [38, 42, 43]. In order to determine the original of these isolates, we compared 28 sequences identified in this study with all available genomic sequences (*n* = 623) of type 2 PRRSV in the GenBank database. The sublineage 10.7 include up to 65 field samples with a wide geographical range, in which the intralineage diversity was the lowest (0.8%), and found nine JXA1-R-like isolates out of the 28 isolates based one the three criteria reported previously [23]. Of course, it was possible that these recombinants might be also derived from other HP-PPRSV vaccine strains, such as HuN4-F112, TJM-F92, and GDr180, because those vaccine strains are derived from HP-PRRSV and there is no published complete sequences of those vaccine strains. The improper use of vaccines may be associated with the emergence of mosaic recombinant strains [44]. Hence, immunization schedules should be optimized to prevent future outbreaks of this infectious disease.

## Conclusion

The findings of this study demonstrated that HP-PRRSV vaccine like strains (JXA1-R-like strains) are widely circulated in China and mosaic recombinant strains associated with the vaccine strains and predominant circulating strains of PRRSV emerged in different provinces of China under current vaccination pressure. PRRSV eradication and control is a pressing situation in China.

## Additional files

Additional file 1: Table S1. Primers used in generating overlapping amplicons spanning PRRSV genomes. (DOCX 13 kb)

Additional file 2: Table S2. Homology between the 28 isolates sequenced in this study. (DOCX 19 kb)

Additional file 3: Table S3. Sequence identity of the JXA1-R-like strains with JXA1 derivatives and representative strains. (DOCX 20 kb)

Additional file 4: Figure S1. Comparisons of partial ORF1a, ORF1b, and GP4 deduced amino acid sequences. *Unique and identical amino acids among the JXA1 derivatives. (PDF 491 kb)

Additional file 5: Figure S2. Phylogenetic analysis of parental regions of putative 10 recombinant strains. The parental vaccine JXA1-R group (sublineage 10.7) is shown in red, the 2009–2010 HP-PRRSV-like group (sublineage 10.6) is shown in deep sky blue, and the NADC30-like strain (lineage 9) is shown in green. Putative recombinant strains are labeled with black triangles (▲). (PDF 502 kb)

Additional file 6: Figure S3. Morbidity and mortality rates among farms with pigs infected with recombinant JXA1-R-like strains. (PDF 87 kb)

## Abbreviations

HP-PRRSV: Highly pathogenic porcine reproductive and respiratory syndrome virus
IHC: Immunohistochemistry
MLVs: Modified live-attenuated vaccines
ORFs: Open reading frames
PCR: Polymerase chain reaction
PRRS: Porcine reproductive and respiratory syndrome
PRRSV: Porcine reproductive and respiratory syndrome virus
RT-PCR: Reverse transcription polymerase chain reaction
