# Supplementary material for: Emergence of mosaic recombinant strains potentially associated with vaccine JXA1-R and predominant circulating strains of porcine reproductive and respiratory syndrome virus in different provinces of China
Source: Virol J. 2017 Apr 4;14:67. doi: 10.1186/s12985-017-0735-3 (PMC5379541; doi:10.1186/s12985-017-0735-3)
Supplement: Supplementary file 1 — Primers used in generating overlapping amplicons spanning PRRSV genomes. (DOCX 13 kb) [file 12985_2017_735_MOESM1_ESM.docx]

### Additional file 1: Table S1. Primers used in generating overlapping amplicons spanning PRRSV genomes.

*Annealing positions based on strain VR-2332 (accession number U87392).

| Primer | Genomic position* | Sequence |
| --- | --- | --- |
| 1F | 1-22 | 5' ATGACGTATAGGTGTTGGCTCT 3' |
| 1R | 1552-1573 | 5' CTCTTTCRGGRAGGGTGGTYTC 3' |
| 2F | 1495-1511 | 5' AAYTGTGGTTGGCACTG 3' |
| 2R | 2185-2202 | 5' CCAATCAAARGAGGTGTC 3' |
| 3F | 2130-2150 | 5' AAGTCTTGARGAATGCTTGGC 3' |
| 3R | 3154-3169 | 5' CCGAGRYTTCRCTCAG 3' |
| 4F | 3043-3061 | 5' GAGCYYCTSGATTTGTCTG 3' |
| 4R | 4381-4400 | 5' GTTCTYACACAAGATCCCCA 3' |
| 5F | 4337-4358 | 5' TGGCTGGAGCYTATGTGCTTTC 3' |
| 5R | 5564-5585 | 5' AACARCATGGTRCGACCAGTCA 3' |
| 6F | 5398-5414 | 5' GCCSTGGTGTTRTTGGT 3' |
| 6R | 6819-6839 | 5' AGRAAATCCAAGTCCTCRTCA 3' |
| 7F | 6587-6610 | 5' TGGTYRTGACCTCRCCAGTCCCAG 3' |
| 7R | 8172-8194 | 5' TAAGGTATGTCTCCAAACCTTGT 3' |
| 8F | 8037-8059 | 5' GAGGAAGTTGCACTYAGTGCGCA 3' |
| 8R | 9312-9331 | 5' GAGTCWGTGATRGYTGTCTT 3' |
| 9F | 9174-9190 | 5' GAGGACATGCTYARGGT 3' |
| 9R | 10404-10423 | 5' GGCATRATGTCAAAKACATA 3' |
| 10F | 10299-10318 | 5' GCGTATTGCAATCAYCTTGA 3' |
| 10R | 11676-11692 | 5' GGGTCCAARTACACMGT 3' |
| 11F | 11532-11553 | 5' TGCTGGAAARTGATGTTGGACT 3' |
| 11R | 12840-12859 | 5' GGGCAYACCGTRTAATTCAC 3' |
| 12F | 12445-12461 | 5' GCTGCCTGGAAACAGGT 3' |
| 12R | 13927-13942 | 5' CCATTCAGCTCACATA 3' |
| 13F | 13732-13750 | 5' GGCRACCGTTTTAGCCTGT 3' |
| 13R | 14888-14907 | 5' TGCCGTTGTTATTTGGCATA 3' |
| 14F | 14792-14812 | 5' TCCACTACGGTCAACGGCACA 3' |
| 14R | 15396-Poly (A) tail | 5' TTTTTTAATTDCGGCCGCATGG 3' |
