# Supplementary material for: Emergence of mosaic recombinant strains potentially associated with vaccine JXA1-R and predominant circulating strains of porcine reproductive and respiratory syndrome virus in different provinces of China
Source: Virol J. 2017 Apr 4;14:67. doi: 10.1186/s12985-017-0735-3 (PMC5379541; doi:10.1186/s12985-017-0735-3)
Supplement: Supplementary file 2 — Homology between the 28 isolates sequenced in this study. (DOCX 19 kb) [file 12985_2017_735_MOESM2_ESM.docx]

###### Additional file 2: Table S2. Homology between the 28 isolates sequenced in this study.

| **No.** | **Lineage/ Sublineage** | **Name** | **% Homology** | | | | | | | | | | | | | | | | | | | | | | | | | | | |
| --- | --- | --- | --- | --- | --- | --- | --- | --- | --- | --- | --- | --- | --- | --- | --- | --- | --- | --- | --- | --- | --- | --- | --- | --- | --- | --- | --- | --- | --- | --- |
|  |  |  | 1 | 2 | 3 | 4 | 5 | 6 | 7 | 8 | 9 | 10 | 11 | 12 | 13 | 14 | 15 | 16 | 17 | 18 | 19 | 20 | 21 | 22 | 23 | 24 | 25 | 26 | 27 | 28 |
| 1 | L9 | 15LN1 |  |  |  |  |  |  |  |  |  |  |  |  |  |  |  |  |  |  |  |  |  |  |  |  |  |  |  |  |
| 2 |  | 15ZJ1 | 94.2 |  |  |  |  |  |  |  |  |  |  |  |  |  |  |  |  |  |  |  |  |  |  |  |  |  |  |  |
| 3 |  | 15HEN4 | 94.3 | 97.5 |  |  |  |  |  |  |  |  |  |  |  |  |  |  |  |  |  |  |  |  |  |  |  |  |  |  |
| 4 |  | 15JX1 | 93.2 | 93.7 | 93.7 |  |  |  |  |  |  |  |  |  |  |  |  |  |  |  |  |  |  |  |  |  |  |  |  |  |
| 5 |  | 15HEN1 | 92.9 | 93.6 | 93.6 | 92.1 |  |  |  |  |  |  |  |  |  |  |  |  |  |  |  |  |  |  |  |  |  |  |  |  |
| 6 |  | 15SC3 | 92.6 | 93.3 | 93.5 | 93.6 | 91.9 |  |  |  |  |  |  |  |  |  |  |  |  |  |  |  |  |  |  |  |  |  |  |  |
| 7 |  | 15LN3 | 91.3 | 91.1 | 91.0 | 91.3 | 92.8 | 92.0 |  |  |  |  |  |  |  |  |  |  |  |  |  |  |  |  |  |  |  |  |  |  |
| 8 | SL10.3 | 15HEB1 | 83.3 | 83.2 | 83.2 | 83.9 | 85.4 | 85.3 | 86.9 |  |  |  |  |  |  |  |  |  |  |  |  |  |  |  |  |  |  |  |  |  |
| 9 | SL10.5 | 15GD4 | 81.4 | 81.5 | 81.4 | 82.3 | 83.6 | 83.7 | 85.2 | 94.4 |  |  |  |  |  |  |  |  |  |  |  |  |  |  |  |  |  |  |  |  |
| 10 |  | 15SN1 | 79.7 | 79.7 | 79.6 | 80.4 | 81.7 | 81.8 | 83.4 | 92.5 | 96.8 |  |  |  |  |  |  |  |  |  |  |  |  |  |  |  |  |  |  |  |
| 11 |  | 15SN2 | 79.7 | 79.8 | 79.7 | 80.5 | 81.7 | 81.9 | 83.5 | 92.5 | 96.7 | 99.5 |  |  |  |  |  |  |  |  |  |  |  |  |  |  |  |  |  |  |
| 12 |  | 15SN3 | 79.5 | 79.5 | 79.5 | 80.3 | 81.5 | 81.6 | 83.3 | 92.3 | 96.5 | 99.3 | 99.5 |  |  |  |  |  |  |  |  |  |  |  |  |  |  |  |  |  |
| 13 | SL10.6 | 15GD1 | 81.0 | 81.2 | 81.0 | 81.9 | 83.1 | 83.1 | 84.7 | 93.7 | 97.1 | 95.3 | 95.3 | 95.0 |  |  |  |  |  |  |  |  |  |  |  |  |  |  |  |  |
| 14 |  | 15GD2 | 81.2 | 81.3 | 81.2 | 82.1 | 83.3 | 83.5 | 85.1 | 94.3 | 97.5 | 95.7 | 95.7 | 95.4 | 99.0 |  |  |  |  |  |  |  |  |  |  |  |  |  |  |  |
| 15 |  | 15GD3 | 81.2 | 81.3 | 81.2 | 82.1 | 83.4 | 83.5 | 85.2 | 94.3 | 97.5 | 95.7 | 95.6 | 95.4 | 99.1 | 99.9 |  |  |  |  |  |  |  |  |  |  |  |  |  |  |
| 16 |  | 15HUN1 | 81.3 | 81.4 | 81.3 | 82.2 | 83.4 | 83.7 | 85.2 | 94.5 | 97.7 | 95.8 | 95.8 | 95.5 | 98.7 | 99.4 | 99.4 |  |  |  |  |  |  |  |  |  |  |  |  |  |
| 17 |  | 15HUN2 | 81.3 | 81.4 | 81.2 | 82.3 | 83.4 | 83.6 | 85.2 | 94.5 | 97.6 | 95.8 | 95.8 | 95.5 | 98.9 | 99.4 | 99.3 | 99.7 |  |  |  |  |  |  |  |  |  |  |  |  |
| 18 |  | 15HEB3 | 81.1 | 81.2 | 81.1 | 81.8 | 83.0 | 83.3 | 84.5 | 93.7 | 96.5 | 94.6 | 94.6 | 94.3 | 96.9 | 97.1 | 97.1 | 97.0 | 97.0 |  |  |  |  |  |  |  |  |  |  |  |
| 19 | SL10.7 | 15JX2 | 81.6 | 81.5 | 81.5 | 82.3 | 83.6 | 83.7 | 85.3 | 95.2 | 98.1 | 96.2 | 96.2 | 95.9 | 97.2 | 97.9 | 97.8 | 98.1 | 98.0 | 96.9 |  |  |  |  |  |  |  |  |  |  |
| 20 |  | 15LN2 | 81.5 | 81.4 | 81.4 | 82.4 | 83.6 | 83.7 | 85.4 | 95.2 | 98.1 | 96.2 | 96.2 | 95.9 | 97.1 | 97.9 | 97.8 | 98.1 | 98.0 | 96.9 | 99.2 |  |  |  |  |  |  |  |  |  |
| 21 |  | 15ZJ2 | 81.5 | 81.5 | 81.5 | 82.3 | 83.6 | 83.8 | 85.4 | 95.3 | 98.3 | 96.4 | 96.4 | 96.1 | 97.4 | 98.1 | 98.0 | 98.3 | 98.2 | 97.1 | 99.4 | 99.3 |  |  |  |  |  |  |  |  |
| 22 |  | 15ZJ3 | 81.6 | 81.6 | 81.6 | 82.5 | 83.7 | 83.9 | 85.5 | 95.3 | 98.3 | 96.4 | 96.4 | 96.1 | 97.3 | 98.1 | 98.0 | 98.3 | 98.2 | 97.1 | 99.4 | 99.3 | 99.6 |  |  |  |  |  |  |  |
| 23 |  | 15JX3 | 81.6 | 81.6 | 81.5 | 82.4 | 83.7 | 83.9 | 85.5 | 95.3 | 98.3 | 96.4 | 96.4 | 96.1 | 97.4 | 98.1 | 98.1 | 98.3 | 98.2 | 97.1 | 99.4 | 99.4 | 99.6 | 99.6 |  |  |  |  |  |  |
| 24 |  | 15SC2 | 81.6 | 81.6 | 81.6 | 82.4 | 83.7 | 83.9 | 85.5 | 95.4 | 98.3 | 96.4 | 96.4 | 96.1 | 97.3 | 98.1 | 98.0 | 98.3 | 98.2 | 97.1 | 99.4 | 99.4 | 99.5 | 99.6 | 99.7 |  |  |  |  |  |
| 25 |  | 15JX4 | 81.5 | 81.5 | 81.5 | 82.4 | 83.6 | 83.8 | 85.4 | 95.3 | 98.3 | 96.4 | 96.4 | 96.1 | 97.3 | 98.1 | 98.0 | 98.4 | 98.2 | 97.0 | 99.4 | 99.3 | 99.5 | 99.5 | 99.6 | 99.6 |  |  |  |  |
| 26 |  | 15SC1 | 81.6 | 81.5 | 81.5 | 82.4 | 83.7 | 83.8 | 85.4 | 95.3 | 98.2 | 96.3 | 96.2 | 96.0 | 97.2 | 97.9 | 97.9 | 98.2 | 98.1 | 96.9 | 99.2 | 99.1 | 99.4 | 99.4 | 99.4 | 99.5 | 99.5 |  |  |  |
| 27 |  | 15HUN3 | 81.5 | 81.5 | 81.5 | 82.4 | 83.6 | 83.8 | 85.4 | 95.2 | 98.2 | 96.3 | 96.3 | 96.0 | 97.2 | 98.0 | 97.9 | 98.2 | 98.1 | 97.0 | 99.3 | 99.2 | 99.4 | 99.4 | 99.5 | 99.4 | 99.4 | 99.3 |  |  |
| 28 |  | 15HEN3 | 81.4 | 81.4 | 81.3 | 82.2 | 83.6 | 83.6 | 85.2 | 95.2 | 98.0 | 96.1 | 96.0 | 95.8 | 97.0 | 97.7 | 97.7 | 98.0 | 97.9 | 96.8 | 99.0 | 99.0 | 99.2 | 99.2 | 99.2 | 99.2 | 99.2 | 99.1 | 99.1 |  |
