# Supplementary material for: Emergence of mosaic recombinant strains potentially associated with vaccine JXA1-R and predominant circulating strains of porcine reproductive and respiratory syndrome virus in different provinces of China
Source: Virol J. 2017 Apr 4;14:67. doi: 10.1186/s12985-017-0735-3 (PMC5379541; doi:10.1186/s12985-017-0735-3)
Supplement: Supplementary file 3 — Sequence identity of the JXA1-R-like strains with JXA1 derivatives and representative strains. (DOCX 20 kb) [file 12985_2017_735_MOESM3_ESM.docx]

###### Additional file 3: Table S3. Sequence identity of the JXA1-R-like strains with JXA1 derivatives and representative strains.

|  | **VR-2332** | **CH-1a** | **JXA1** | **JXA1 P10** | **JXA1 P15** | **JXA1 P45** | **JXA1 P70** | **JXA1 P80** | **JXA1-P100** | **JXA1-P110** | **JXA1-P120** | **JXA1-P130** | **JXA1-P140** | **JXA1-P150** | **JXA1-P160** | **JXA1-P170** |
| --- | --- | --- | --- | --- | --- | --- | --- | --- | --- | --- | --- | --- | --- | --- | --- | --- |
| 11NZ-GD | 89.94 | 94.93 | 99.06 | 99.43 | 99.51 | 99.56 | 99.48 | 99.44 | 99.52 | 99.50 | 99.50 | 99.48 | 99.46 | 99.46 | 99.44 | 99.45 |
| 11SH1-GD | 89.67 | 94.96 | 99.04 | 99.34 | 99.42 | 99.46 | 99.41 | 99.40 | 99.46 | 99.43 | 99.43 | 99.42 | 99.40 | 99.39 | 99.33 | 99.37 |
| 11SH-GD | 89.95 | 94.89 | 98.88 | 99.19 | 99.26 | 99.28 | 99.25 | 99.26 | 99.33 | 99.33 | 99.32 | 99.33 | 99.30 | 99.31 | 99.25 | 99.28 |
| 11XX-GD | 89.95 | 94.95 | 99.08 | 99.43 | 99.52 | 99.57 | 99.48 | 99.45 | 99.52 | 99.51 | 99.50 | 99.49 | 99.47 | 99.47 | 99.45 | 99.45 |
| NT1 | 89.74 | 95.08 | 99.21 | 99.56 | 99.65 | 99.69 | 99.62 | 99.62 | 99.68 | 99.67 | 99.69 | 99.66 | 99.67 | 99.64 | 99.63 | 99.63 |
| NT2 | 89.95 | 95.09 | 99.22 | 99.52 | 99.61 | 99.65 | 99.63 | 99.61 | 99.69 | 99.69 | 99.65 | 99.67 | 99.63 | 99.63 | 99.60 | 99.61 |
| NT3 | 89.95 | 95.10 | 99.22 | 99.55 | 99.62 | 99.63 | 99.62 | 99.58 | 99.65 | 99.65 | 99.63 | 99.63 | 99.61 | 99.59 | 99.58 | 99.58 |
| 15HEN3 | 89.58 | 94.78 | 98.87 | 99.24 | 99.32 | 99.32 | 99.21 | 99.22 | 99.30 | 99.28 | 99.27 | 99.27 | 99.25 | 99.24 | 99.25 | 99.22 |
| 15HUN3 | 89.84 | 95.02 | 99.12 | 99.48 | 99.54 | 99.57 | 99.47 | 99.46 | 99.50 | 99.50 | 99.49 | 99.50 | 99.47 | 99.47 | 99.45 | 99.45 |
| 15JX2 | 89.60 | 94.91 | 99.07 | 99.39 | 99.48 | 99.53 | 99.42 | 99.43 | 99.48 | 99.51 | 99.50 | 99.48 | 99.48 | 99.46 | 99.43 | 99.45 |
| 15JX3 | 89.74 | 95.08 | 99.26 | 99.62 | 99.71 | 99.77 | 99.66 | 99.65 | 99.71 | 99.69 | 99.69 | 99.68 | 99.66 | 99.65 | 99.62 | 99.64 |
| 15JX4 | 89.73 | 95.08 | 99.22 | 99.55 | 99.63 | 99.69 | 99.62 | 99.61 | 99.67 | 99.65 | 99.66 | 99.66 | 99.63 | 99.63 | 99.59 | 99.62 |
| 15LN2 | 89.84 | 94.86 | 99.05 | 99.33 | 99.42 | 99.45 | 99.39 | 99.37 | 99.44 | 99.45 | 99.43 | 99.42 | 99.43 | 99.41 | 99.37 | 99.40 |
| 15SC1 | 89.70 | 95.04 | 99.09 | 99.43 | 99.50 | 99.54 | 99.48 | 99.47 | 99.54 | 99.52 | 99.52 | 99.53 | 99.48 | 99.51 | 99.47 | 99.48 |
| 15SC2 | 89.95 | 95.05 | 99.25 | 99.55 | 99.63 | 99.71 | 99.65 | 99.65 | 99.72 | 99.68 | 99.67 | 99.69 | 99.65 | 99.64 | 99.59 | 99.63 |
| 15ZJ2 | 89.68 | 95.07 | 99.28 | 99.58 | 99.65 | 99.69 | 99.58 | 99.54 | 99.64 | 99.60 | 99.61 | 99.62 | 99.58 | 99.55 | 99.56 | 99.56 |
| 15ZJ3 | 89.98 | 95.03 | 99.21 | 99.56 | 99.65 | 99.69 | 99.62 | 99.61 | 99.67 | 99.65 | 99.64 | 99.65 | 99.62 | 99.62 | 99.60 | 99.60 |
| GX1001 | 89.57 | 94.91 | 98.93 | 99.25 | 99.32 | 99.36 | 99.26 | 99.26 | 99.33 | 99.35 | 99.37 | 99.35 | 99.34 | 99.32 | 99.29 | 99.31 |
| GX1002 | 89.62 | 95.01 | 99.18 | 99.48 | 99.57 | 99.65 | 99.66 | 99.66 | 99.72 | 99.71 | 99.69 | 99.70 | 99.68 | 99.68 | 99.61 | 99.66 |
| GX1003 | 89.73 | 95.15 | 99.32 | 99.69 | 99.77 | 99.80 | 99.71 | 99.69 | 99.76 | 99.77 | 99.79 | 99.77 | 99.76 | 99.73 | 99.71 | 99.73 |
| HB2014001 | 89.84 | 94.85 | 99.21 | 99.21 | 99.28 | 99.32 | 99.24 | 99.23 | 99.30 | 99.34 | 99.35 | 99.34 | 99.32 | 99.30 | 99.31 | 99.29 |
| HEB 20130008-14 | 89.69 | 95.05 | 99.21 | 99.53 | 99.59 | 99.59 | 99.54 | 99.56 | 99.63 | 99.63 | 99.61 | 99.61 | 99.60 | 99.58 | 99.54 | 99.55 |
| HEB-2013 | 89.66 | 95.07 | 99.22 | 99.54 | 99.61 | 99.62 | 99.51 | 99.51 | 99.58 | 99.56 | 99.58 | 99.55 | 99.54 | 99.52 | 99.49 | 99.51 |
| HENPDS-2 | 89.76 | 95.12 | 99.35 | 99.65 | 99.73 | 99.78 | 99.76 | 99.73 | 99.78 | 99.80 | 99.78 | 99.80 | 99.76 | 99.77 | 99.71 | 99.74 |
| HENZK-1 | 89.58 | 94.92 | 99.04 | 99.41 | 99.50 | 99.50 | 99.37 | 99.35 | 99.41 | 99.45 | 99.46 | 99.43 | 99.44 | 99.40 | 99.39 | 99.41 |
| HNxa14 | 89.58 | 94.96 | 99.15 | 99.46 | 99.54 | 99.62 | 99.58 | 99.56 | 99.63 | 99.63 | 99.62 | 99.63 | 99.60 | 99.57 | 99.56 | 99.56 |
| HNyc13 | 89.79 | 94.92 | 99.01 | 99.36 | 99.45 | 99.50 | 99.37 | 99.37 | 99.45 | 99.45 | 99.45 | 99.45 | 99.42 | 99.41 | 99.41 | 99.40 |
| HUN-2014 | 89.42 | 94.75 | 98.80 | 99.10 | 99.18 | 99.18 | 99.15 | 99.13 | 99.21 | 99.22 | 99.24 | 99.23 | 99.22 | 99.20 | 99.16 | 99.18 |
| JL-04 12 | 89.85 | 94.88 | 99.07 | 99.35 | 99.42 | 99.43 | 99.41 | 99.39 | 99.46 | 99.43 | 99.41 | 99.42 | 99.39 | 99.39 | 99.35 | 99.35 |
| NVDC-13SXJC-2014 | 89.62 | 94.96 | 99.15 | 99.45 | 99.52 | 99.54 | 99.47 | 99.47 | 99.54 | 99.54 | 99.52 | 99.51 | 99.52 | 99.51 | 99.45 | 99.49 |
| NVDC-BJ3-2012 | 89.59 | 94.81 | 98.95 | 99.24 | 99.32 | 99.35 | 99.36 | 99.36 | 99.43 | 99.41 | 99.40 | 99.41 | 99.38 | 99.37 | 99.34 | 99.33 |
| NVDC-BJ4-2012 | 89.54 | 94.83 | 98.85 | 99.20 | 99.28 | 99.25 | 99.14 | 99.14 | 99.22 | 99.20 | 99.22 | 99.20 | 99.19 | 99.16 | 99.15 | 99.16 |
| NVDC-BJ5-2012 | 89.51 | 94.87 | 99.03 | 99.24 | 99.28 | 99.26 | 99.13 | 99.13 | 99.21 | 99.20 | 99.22 | 99.19 | 99.18 | 99.16 | 99.14 | 99.15 |
| NVDC-BJ6-2012 | 89.55 | 94.94 | 98.96 | 99.33 | 99.40 | 99.37 | 99.26 | 99.26 | 99.34 | 99.33 | 99.35 | 99.32 | 99.31 | 99.29 | 99.28 | 99.28 |
| NVDC-BJ9-2012 | 89.58 | 94.96 | 99.06 | 99.41 | 99.50 | 99.55 | 99.47 | 99.44 | 99.49 | 99.50 | 99.50 | 99.52 | 99.46 | 99.46 | 99.44 | 99.46 |
| NVDC-BJPG-2013 | 89.50 | 94.87 | 98.83 | 99.15 | 99.24 | 99.30 | 99.16 | 99.16 | 99.22 | 99.20 | 99.20 | 99.20 | 99.20 | 99.17 | 99.14 | 99.16 |
| NVDC-HBCZ-2013 | 89.51 | 94.70 | 98.80 | 99.11 | 99.16 | 99.18 | 99.09 | 99.05 | 99.12 | 99.13 | 99.13 | 99.13 | 99.11 | 99.08 | 99.08 | 99.07 |
| NVDC-HeB1-2011 | 89.89 | 94.97 | 99.12 | 99.43 | 99.50 | 99.49 | 99.47 | 99.47 | 99.52 | 99.50 | 99.50 | 99.51 | 99.46 | 99.48 | 99.43 | 99.46 |
| NVDC-HuNCS-2014 | 89.60 | 94.00 | 99.16 | 99.48 | 99.54 | 99.58 | 99.50 | 99.48 | 99.57 | 99.53 | 99.56 | 99.54 | 99.52 | 99.49 | 99.47 | 99.48 |
| NVDC-SD2-2012 | 89.61 | 94.90 | 99.03 | 99.39 | 99.47 | 99.52 | 99.49 | 99.47 | 99.52 | 99.50 | 99.48 | 99.51 | 99.46 | 99.48 | 99.45 | 99.45 |
| NVDC-SDXX-2013 | 89.77 | 94.80 | 98.87 | 99.22 | 99.29 | 99.31 | 99.23 | 99.22 | 99.30 | 99.28 | 99.26 | 99.26 | 99.25 | 99.24 | 99.24 | 99.22 |
| NVDC-shh01-2014 | 89.86 | 94.93 | 99.07 | 99.36 | 99.42 | 99.43 | 99.39 | 99.41 | 99.50 | 99.48 | 99.45 | 99.46 | 99.45 | 99.43 | 99.38 | 99.40 |
| NVDC-SHH02-2014 | 89.66 | 94.62 | 98.69 | 98.98 | 99.03 | 99.06 | 99.01 | 99.02 | 99.10 | 99.07 | 99.05 | 99.07 | 99.05 | 99.03 | 98.99 | 99.00 |
| NVDC-SXJC-2013 | 89.62 | 94.96 | 99.15 | 99.45 | 99.52 | 99.54 | 99.47 | 99.47 | 99.54 | 99.54 | 99.52 | 99.51 | 99.52 | 99.51 | 99.45 | 99.49 |
