# Supplementary material for: Emergence of mosaic recombinant strains potentially associated with vaccine JXA1-R and predominant circulating strains of porcine reproductive and respiratory syndrome virus in different provinces of China
Source: Virol J. 2017 Apr 4;14:67. doi: 10.1186/s12985-017-0735-3 (PMC5379541; doi:10.1186/s12985-017-0735-3)
Supplement: Supplementary file 4 — Comparisons of partial ORF1a, ORF1b, and GP4 deduced amino acid sequences. *Unique and identical amino acids among the JXA1 derivatives. (PDF 491 kb) [file 12985_2017_735_MOESM4_ESM.pdf]

	ORF1a							ORF1b			GP4	Total
	684	780	955	981	1091	1420	1658	418	842	1030	170	
VR-2332	T I K L P	K L A N	D L S A S	E G H E A E E	G R L K F	T R S S L	Q E I A L	A E E H L	P S R T G	V N L A V	C L F A I	
CH-1a	. T K Q L	. . . .	. . . . F	. . Q . A . .	. . S G .	. . . . .	. . . . .	. . . . .	. . . . .	. . . . .	. . . . .	0
JXA1	. T . Q .	. . . .	. . . . .	. . Q . V . .	. . F E .	. . . . .	. . . . .	. . . . .	. . . . .	. . . . .	. . L P S	0
JXA1 P10	. T D Q .	. . T .	. . . . .	R . Q . V . G	. . F E .	. . L . .	. . . . .	. . . . .	. . H . .	. . . T .	. . . . .	7
JXA1 P15	. T D Q .	. . T .	. . . . .	R . Q . V . G	. M F E .	. . L . .	. . . . .	. . G . .	. . H . .	. . . T .	. . . . .	9
JXA1 P45	. T D Q .	. . T .	. . . . .	R . Q . V . G	. M F E .	. . L . .	. . . . .	. . G . .	. . H . .	. . . T .	. . V . .	10
JXA1 P70	. T D Q .	. . T .	. . . V .	R . Q . V . G	. M F E .	. . L . .	. D . . .	. . G . .	. . H . .	. . . T .	. . V . .	12
JXA1 P80	. T D Q .	. . T .	. . . V .	R . Q . V . G	. M F E .	. . L . .	. D . . .	. . G . .	. . H . .	. . . T .	. . V . .	12
JXA1-P100	. T D Q .	. . T .	. . . V .	R . Q . V . G	. M F E .	. . L . .	. D . . .	. . G . .	. . H . .	. . . T .	. . V . .	12
JXA1-P110	. T D Q .	. . T .	. . . V .	R . Q . V . G	. M F E .	. . L . .	. D . . .	. . G . .	. . H . .	. . . T .	. . . . .	11
JXA1-P120	. T D Q .	Q . T .	. . . V .	R . Q . V . G	. M F E .	. . L . .	. D . . .	. . G . .	. . H . .	. . . T .	. . . . .	11
JXA1-P130	. T D Q .	E . T .	. . . V .	R . Q . V . G	. M F E .	. . L . .	. D . . .	. . G . .	. . H . .	. . . T .	. . . . .	11
JXA1-P140	. T D Q .	E . T .	. . . V .	R . Q . V . G	. M F E .	. . L . .	. D . . .	. . G . .	. . H . .	. . . T .	. . . . .	11
JXA1-P150	. T D Q .	E . T .	. . . V .	R . Q . V . G	. M F E .	. . L . .	. D . . .	. . G . .	. . H . .	. . . T .	. . . . .	11
JXA1-P160	. T D Q .	E . T .	. . . V .	R . Q . V . G	. M F E .	. . L . .	. D . . .	. . G . .	. . H . .	. . . T .	. . . . .	11
JXA1-P170	. T D Q .	E . T .	. . . V .	R . Q . V . G	. M F E .	. . L . .	. D . . .	. . G . .	. . H . .	. . . T .	. . . . .	11
11NZ-GD	. T D Q .	. . T .	. . . V .	R . Q . V . G	. M F E .	. . L . .	. D . . .	. . G . .	. . H . .	. . . T .	. . V . .	12
11SH1-GD	. T D Q .	. . T .	. . . V .	R . Q . V . .	. K F E .	. . L . .	. . . . .	. . G . .	. . H . .	. . . . .	. . V . .	8
11SH-GD	. T D Q .	. . T .	. . . V .	R . Q . V . .	. . F E .	. . L . .	. . . . .	. . G . .	. . H . .	. . . . .	. . . . .	7
11XX-GD	. T D Q .	. . T .	. . . V .	R . Q . V . G	. M F E .	. . L . .	. D . . .	. . G . .	. . H . .	. . . T .	. . V . .	12
NT1	. T D Q .	. . T .	. . . V .	R . Q . V . .	. M F E .	. . L . .	. D . . .	. . G . .	. . H . .	. . . T .	. . V . .	11
NT2	. T D Q .	. . T .	. . . V .	R . Q . V . G	. M S E .	. . L . .	. D . . .	. . G . .	. . H . .	. . . . .	. . . . .	10
NT3	. T D Q .	. . T .	. . . V .	R . Q . V . G	. . S E .	. . L . .	. D . . .	. . G . .	. . H . .	. . . T .	. . V . .	11
15HEN3	. T D Q .	. . T .	. . . V .	R . Q . D . G	. K S E .	. . L . .	. . . . .	. . G . .	. . H . .	. . . . .	. . . . .	8
15HUN3	A T D Q .	. . T .	. . . V .	R . Q . V . G	. K F E .	. . L . .	. . . . .	. . G . .	. . H . .	. . . . .	. . . . .	8
15JX2	. T D Q .	. . . .	. . . V .	R . Q . V . .	. M F E .	. . L . .	. D . . .	. . G . .	. . H . .	. . . T .	. . V . .	10
15JX3	. T D Q .	. . T .	. . . V .	R . Q . V . G	. M F E .	. . L . .	. . . . .	. . G . .	. . H . .	. . . T .	. . V . .	11
15JX4	. T D Q .	. . T .	. . . V .	R . Q . V . .	. M F E .	. . . . .	. . . . .	. . G . .	. . H . .	. . . . .	. . . . .	7
15LN2	. T D Q .	. . T .	. . . . .	R . Q . V . .	. M F E .	. . L . .	. D . . .	. . G . .	. . H . .	. . . . .	. F V . .	9
15SC1	. T D P .	. . T .	. . . V .	R . Q . V . .	. K F E .	. . L . .	. . . . .	. . G . .	. . H . .	. . . . .	. . . . .	7
15SC2	. T D Q .	. . T .	. . . V .	R . Q . V . .	. M F E .	. . L . .	. D . . .	. . G . .	. . H . .	. . . . .	. . V . .	10
15ZJ2	. T D Q .	. . . .	. . . V .	R . Q . V . G	. M F E .	. . L . .	. . V . .	. . G . .	. . H . .	. . . T .	. . V . .	10
15ZJ3	. T D Q .	. . . .	. . . V .	R . Q . V . G	. M F E .	. . L . .	. D . . .	. . G . .	. . H . .	. . . T .	. . V . .	11
GX1001	. T E Q .	. . . .	. . . V .	R . Q . V . G	. M F E .	. . L . .	. . V . .	. . G . .	. . H . .	. . . T .	. . . . .	8
GX1002	. T D Q .	. . T .	. . . V .	R . Q . V . G	. M F E .	. . L . .	. D . . .	. . G . .	. . H . .	. . . T .	. . . . .	11
GX1003	. T D Q .	. . T .	. . . V .	R . Q . V . G	. K F E .	. . L . .	. . V . .	. . G . .	. . H . .	. . . T .	. . . . .	9
HB2014001	. T D Q .	. . T .	. . . V .	S . Q . V . .	. M F E .	. . . . .	. D . . .	. . G . .	. . H . .	. . . . .	. . . . .	7
HEB 20130008-14	. T D Q .	. . T .	. . . V .	R . Q . V . G	. . F E .	. . L . .	. D . . .	. . G . .	. . H . .	. . . . .	. . . . .	9
HEB-2013	A T D Q .	. . T .	. . . V .	R . Q . V . .	. . S E .	. . L . .	. D . . .	. . G . .	. . H . .	. . . . .	. . I . .	8
HENPDS-2	. T D Q .	. . T .	. . . V .	R . Q . V . G	. M F E .	. . L . .	. . . . .	. . G . .	. . H . .	. . . T .	. . V . .	11
HENZK-1	. T D Q .	. . I .	. . . . .	R . Q . V . .	. M S E .	. . L . .	. . . . .	. . G . .	. . H . .	. . . T .	. . . . .	7
HNxa14	. T D Q .	. . I .	. . . V .	R . Q . V . G	. M F E .	. . L . .	. . . . .	. . G . .	. . H . .	. . . T .	. . V . .	10
HNyc13	. T D H .	. . T .	. . . V .	R . Q . V . G	. M . E .	. . L . .	. . . . .	. . G . .	. . H . .	. . . T .	. I V . .	11
HUN-2014	. T D Q .	. . T .	. . . V .	R . Q . V . G	. T F E .	. . . . .	. . . . .	. . G . .	. . H . .	. . . . .	. . . . .	7
JL-04/12	. T D Q .	. . T .	. . . V .	S . Q . V . .	. . F E .	. . L . .	. D . . .	. . G . .	. . H . .	. . . . .	. . V . .	8
NVDC-13SXJC-2014	. T D Q .	. . I .	. . . V .	R . Q . V . .	. . F E .	. . . . .	. . . . .	. . G . .	. . H . .	. . . T .	. . V . .	7
NVDC-BJ3-2012	. T D Q .	N . T .	. . . V .	R . Q . V . G	. K F E .	. . . . .	. D . . .	. . G . .	. . H . .	. . . . .	. . . . .	8
NVDC-BJ4-2012	. T D Q .	. . T .	. . . V .	R . Q . V . G	. . S E .	. . . . .	. D . . .	. . G . .	. . H . .	. . . . .	. . . . .	8
NVDC-BJ5-2012	. T D Q .	. . T .	. . . V .	R . Q . V . G	. . S E .	. . . . .	. D . . .	. . G . .	. . H . .	. . . . .	. . . . .	8
NVDC-BJ6-2012	. T D Q .	. . T .	. . . V .	R . Q . V . G	. . S E .	. . L . .	. D . . .	. . G . .	. . H . .	. . . . .	. . . . .	9
NVDC-BJ9-2012	. T D Q .	. . I .	. . . V .	R . Q . V . G	. M F E .	. . L . .	. . . . .	. . G . .	. . H . .	. . . . .	. . V . .	9
NVDC-BJPG-2013	I T D Q .	. . . .	. . . V .	R . Q . V . G	. M S E .	. . . . .	. . . . .	. . G . .	. . H . .	. . . T .	. . V . .	9
NVDC-HBCZ-2013	. T D Q .	. . T .	. . . V .	R . Q . V . .	. . F E .	. . L . .	. . . . .	. . G . .	. . . . .	. . . . .	. . . . .	6
NVDC-HeB1-2011	. T D Q .	. . T .	. . . V .	R . Q . V . G	. . F E .	. . L . .	. D . . .	. . G . .	. . H . .	. . . . M	. . L P S	9
NVDC-HuNCS-2014	. T D Q .	. . T .	. . . V .	R . Q G V . G	. M F E .	. . L . .	. . . . .	. . G . .	. . H . .	. . . T .	. . V . .	11
NVDC-SD2-2012	. T D Q .	. . I .	. . . V .	R . Q . V . G	. M F E .	. . L . .	. D . . .	. . G . .	. . H . .	. . . . .	. . V . .	10
NVDC-SDXX-2013	. T D Q .	. . I .	. . . V .	R . Q . V . G	. K F E .	. . L . .	. D . . .	. . . . .	. . H . .	. . . T .	. . . . .	8
NVDC-shh01-2014	. T D Q .	. . I .	. . . V .	R . Q . V . .	. M S E .	. . . . .	. . . . .	. . . . .	. . H . .	. . I . .	. . V . .	6
NVDC-SHH02-2014	. T D Q .	. . T .	. . . V .	R . Q . V . .	. M F E .	. . L . .	. . . . .	. . . . .	. . H . .	. . I . .	. . . . .	7
NVDC-SXJC-2013	. T D Q .	. . I .	. . . V .	R . Q . V . .	. . F E .	. . . . .	. . . . .	. . G . .	. . H . .	. . . T .	. . V . .	7
	Asp686	Thr782	Val958	Arg981, Gly987	Met1092	Leu1422	Asp1659	Gly420	His844	Thr1033	Val172	
