# Supplementary figures and images for: Emergence of mosaic recombinant strains potentially associated with vaccine JXA1-R and predominant circulating strains of porcine reproductive and respiratory syndrome virus in different provinces of China
Source: Virol J. 2017 Apr 4;14:67. doi: 10.1186/s12985-017-0735-3 (PMC5379541; doi:10.1186/s12985-017-0735-3)

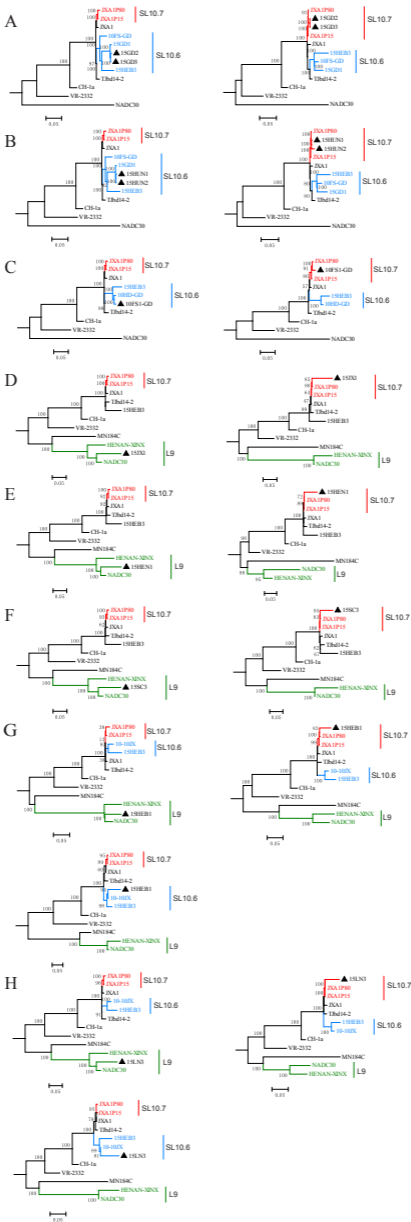

Supplement: Supplementary file 5 — Phylogenetic analysis of parental regions of putative 10 recombinant strains. The parental vaccine JXA1-R group (sublineage 10.7) is shown in red, the 2009–2010 HP-PRRSV-like group (sublineage 10.6) is shown in deep sky blue, and the NADC30-like strain (lineage 9) is shown in green. Putative recombinant strains are labeled with black triangles (▲). (PDF 502 kb) [file 12985_2017_735_MOESM5_ESM.pdf]

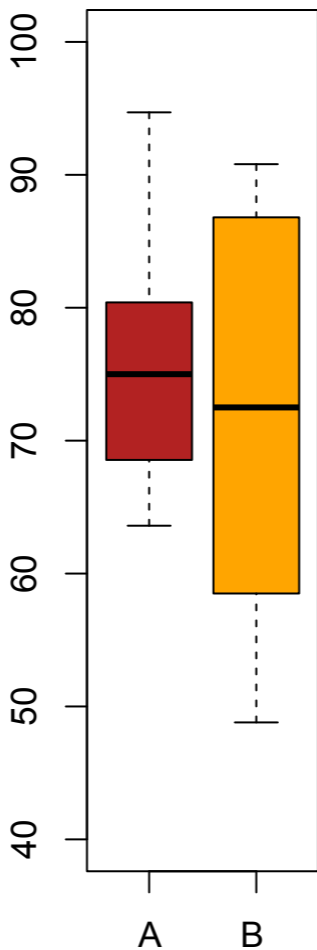

A:morbidity(%), B:mortality(%)

Supplement: Supplementary file 6 — Morbidity and mortality rates among farms with pigs infected with recombinant JXA1-R-like strains. (PDF 87 kb) [file 12985_2017_735_MOESM6_ESM.pdf]
